# RCircos: an R package for Circos 2D track plots

**DOI:** 10.1186/1471-2105-14-244

**Published:** 2013-08-10

**Authors:** Hongen Zhang, Paul Meltzer, Sean Davis

**Affiliations:** 1 Genetics Branch, Center for Cancer Research, National Cancer Institute, National Institutes of Health, Building 37, Room 6138, 37 Convent Drive, Bethesda, MD 20892-4265, USA

**Keywords:** Software, RCircos, R package, Circos, Genomic data visualization

## Abstract

**Background:**

Circos is a Perl language based software package for visualizing similarities and differences of genome structure and positional relationships between genomic intervals. Running Circos requires extra data processing procedures to prepare plot data files and configure files from datasets, which limits its capability of integrating directly with other software tools such as R. Recently published R Bioconductor package ggbio provides a function to display genomic data in circular layout based on multiple other packages, which increases its complexity of usage and decreased the flexibility in integrating with other R pipelines.

**Results:**

We implemented an R package, RCircos, using only R packages that come with R base installation. The package supports Circos 2D data track plots such as scatter, line, histogram, heatmap, tile, connectors, links, and text labels. Each plot is implemented with a specific function and input data for all functions are data frames which can be objects read from text files or generated with other R pipelines.

**Conclusion:**

RCircos package provides a simple and flexible way to make Circos 2D track plots with R and could be easily integrated into other R data processing and graphic manipulation pipelines for presenting large-scale multi-sample genomic research data. It can also serve as a base tool to generate complex Circos images.

## Background

Circos is a Perl language based software package for visualizing similarities and differences of genome structure and positional relationships between genomic intervals [1]. Although many tools for genomic data visualization have been developed [2-5], Circos is commonly used by the genome research community to present large-scale multi-sample genomic research data (http://circos.ca/in_literature/scientific/). While Circos is powerful and flexible in displaying genomic data it requires extra data procedures to prepare plot data files and configuration files from datasets, which limits its capability of integrating directly with other software tools such as R, one of the most commonly used toolsets in processing and statistical analysis of genomic data.

Recently, Yin et al. [6] published a Bioconductor package, ggbio, that includes a function to display genomic data in a circular layout and covers many of the basic Circos-like plots. The ggbio package relies on multiple other packages and offers some integration with other Bioconductor packages. However, ggbio is somewhat complex (but powerful) and relies on high-level plotting packages. RCircos was developed as a simple and flexible approach to Circos-like plots that uses base R graphics.

To make Circos 2D track plots simple and flexible, we implemented an R package, RCircos, that relies on base graphics and R data structures. With RCircos, Circos 2D track plots could be easily generated and the procedures can be effectively integrated with other R pipelines including graphics output manipulation.

## Implementation

Packages used to build RCircos are all included in the R base installation (http://www.r-project.org/). Graphics functionality is accomplished using base R graphics. No other package is required unless input data is associated with special data structure such as GenomicRanges objects and need to be processed separately.

To reduce the complexity of the usage, all functions in RCircos use a simple data frame as input. The first three columns of the data frame are genomic position data in the order of chromosome name, start position, and end position followed by one or more data columns except of link data which requires paired chromosome positions for each row. Data set in data frame is directly passed to the plot function without need of further processing. Sample data are included in the package to show the input data formats and can be easily explored with data(package = “RCircos”) function.

We follow the layout paradigm set forth by Circos and arrange data plots by tracks. The core track is the chromosome ideogram track with highlighting and labels. Data plot tracks can be placed inside or outside of chromosome ideogram track. A set of parameters is used to control the plot pattern such as chromosome width, number of base pairs per chromosome unit, track height, and point type. These parameters are initialized prior to plotting but can be customized to meet the requirements of different plot types.

RCircos is designed such that each type of Circos 2D track plot is drawn with a separate and dedicated function call. To make RCircos more flexible in integrating with other R pipelines, we chose low level plot functions of R including points(), lines(), polygon(), and text() to implement graphic plot functions of RCircos. All RCircos plots work on an existing plot facilitating plot customization using standard R plot functionality.

## Result and discussion

RCircos implements most of Circos 2D track plots including scatter, line, histogram, heatmaps, tiles, connectors, and text labels. We use the chromosome ideogram tables from UCSC genome browser to generate chromosome ideogram images and currently human, mouse, and rat are available in RCircos, but other species can be supported if relevant ideogram table is provided in a same format as cytoBandIdeo table in the UCSC genome browser [7].

**Figure 1:**
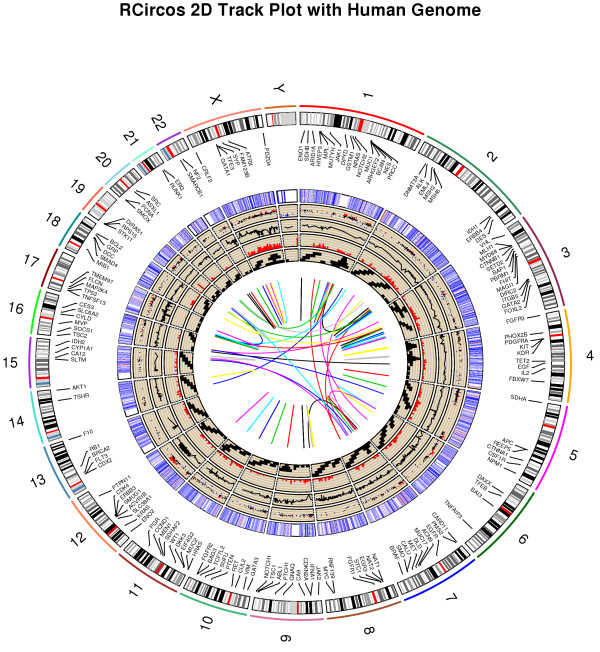
RCircos image showing human chromosome ideogram with data tracks for connectors, gene labels, heatmap, scatter plot, line plot, histogram, tiles, and link lines.

A set of demos and a complete vignette are included in the package to show the RCircos plot procedures for each Circos 2D track plot type. Figure 1 was generated using the code below with build-in datasets and default parameters showing the human chromosome ideogram track along with data tracks for connectors, gene labels, heatmap, scatter plot, line plot, histogram, tiles, and link lines.

**Figure 2:**
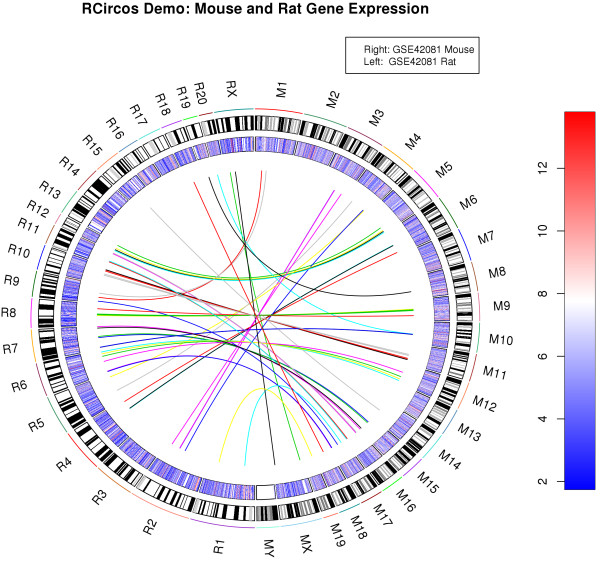
**Combination of RCircos plot and other R graphics plot.** Mouse and rat chromosome ideograms, heatmaps, and link lines are drawn with RCircos with two input datasets. Title, legend, and color key are added with function calls of R graphics package.

Since we implemented the RCircos plots with base R graphics, combining RCircos with other R plot functions is straightforward. Figure 2 show a heatmap generated with demo (“R.Circos.Demo.Mouse.And.Rat”) with blue and red colors for comparison of gene expression between mouse and rat (GEO data accession number: GSE42081) and link lines between top 50 highly expressed genes in mouse and the same genes in rat. Legend and color key for the heatmap were added with the legend() and image() functions.

## Conclusions

The RCircos package provides simple and flexible functionality to generate Circos 2D track plots with R and can be easily integrated into other R data processing and graphic manipulation pipelines to present large-scale multi-sample genomic research data.

## Availability and requirements

The package and source code of RCircos are available for download and install from CRAN website (http://www.r-project.org) with the license of GPL (> = 2).

## Competing interests

The authors declare that they have no competing interests.

## Author’s contributions

HZ designed and implemented the software package, and wrote manuscript. SD participated in the software design and drafted the manuscript. PM revised the manuscript critically. All authors read and approved the final manuscript.
